# Liquid–Liquid Interface-Based Thiocyanate Surface Treatment for Bright and Stable CsPbBr_3_ Nanocrystals

**DOI:** 10.1021/acs.chemmater.5c00803

**Published:** 2025-05-25

**Authors:** Rachel Lifer, Nathan Rafisiman, Saar Shaek, Arghyadeep Basu, Yaron Kauffmann, Nicholas G. Pavlopoulos, Ivano E. Castelli, Lev Chuntonov, Yehonadav Bekenstein

**Affiliations:** † Department of Materials Science and Engineering, Technion − Israel Institute of Technology, 32000 Haifa, Israel; ‡ Department of Chemistry, Technion − Israel Institute of Technology, 32000 Haifa, Israel; § The Solid-State Institute, Technion − Israel Institute of Technology, 32000 Haifa, Israel; ∥ Department of Energy Conversion and Storage (DTU Energy), Technical University of Denmark, Anker Engelunds Vej 301, 2800 Kongens Lyngby, Denmark; ⊥ The Resnick Sustainability Center for Catalysis, Technion − Israel Institute of Technology, Technion City, 3200009 Haifa, Israel

## Abstract

Enhancing the efficiency and stability of lead halide perovskite devices is crucial to their practical application. Previous treatments with thiocyanate (SCN^–^) have demonstrated significant improvements in the photoluminescence quantum yield (PLQY) and stability of CsPbBr_3_ nanocrystals (NCs), but the underlying mechanisms remain partially unresolved. Addressing the challenge of low SCN^–^ solubility in traditional nonpolar solvents, our study introduces a urea-ammonium thiocyanate (UAT)-based ionic liquid surface treatment. This method facilitates a higher SCN^–^ loading by creating a liquid–liquid interface that is compatible with the organic colloidal suspension, preventing NC degradation, and achieving near-unity PLQY. Utilizing transmission electron microscopy techniques, we present atomic resolution evidence that thiocyanate-treated surfaces are rich in sulfur and display structural dilation of the lattice spacing of 3%. This supports that thiocyanate acts as a pseudohalide and binds to Pb cations on the NC surfaces. As a result, the treated NCs show enhanced stability against ionic substitution while maintaining the perovskite structure intact. Our findings provide conclusive evidence that the primary mechanism of performance enhancement is the passivation of surface traps attributed to bromide vacancies rather than the scavenging of excess lead cation. This surface treatment method slows ion migration, a prominent challenge in photovoltaics, offering a significant advancement in the development of perovskite-based devices.

## Graphical abstract

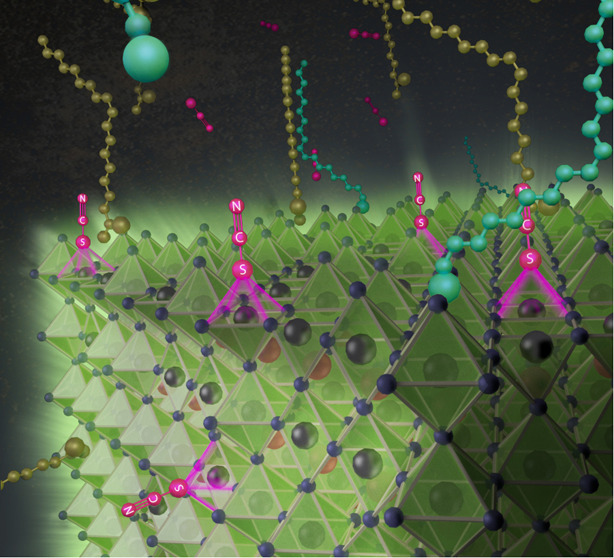

## Introduction

Metal halide perovskites have been gaining interest in recent years in optoelectronic applications.^1–4^ As colloidal nanoparticles, their surface chemistries can be used to tailor their properties and aid integration into devices, including solar cells, LEDs, and sensors.^5^ Their excellent properties, high photoluminescence quantum yield (PLQY), and tunable band gap have made them popular options for these applications. Their defect tolerance makes them robust, but even then, they are not defect-free.^6,7^

Many issues that plague perovskite solar cells also present themselves in their nanocrystalline counterparts. The most prevalent defects would arise on the surface as Shockley–Read–Hall shallow trapped states, typically lead or bromide deficiencies.^8^ These traps decrease the PLQY of the nanocrystals and, subsequently, the power conversion efficiency of their solar cell counterparts. Different passivation strategies have been employed to improve the NC properties via ligand exchange, inorganic salts,^9–11^ pseudohalides,^12,13^ fluorination,^14^ and surface etching.^15,16^ Several organic ligands have been shown to effectively enhance perovskite NC surface passivation, especially if a tailored headgroup is matched for the passivation of acute defects,^17^ which may result in near-unity PLQY and outstanding long-term stability.^18–21^ Interfacial ligand exchange, wherein surface ligands are replaced at the interface of two immiscible solvents, can play a central role in this process. The use of phase-separated systems, where immiscible solvents form distinct phases, can significantly enhance the flexibility and efficiency of replacing one ligand with another.^22,23^ For example, phase-transfer ligand exchange methods have been demonstrated to facilitate fast and effective surface passivation of quantum dots (QDs), enabling the preparation of highly conductive and optically active films. In these methods, the use of immiscible solvents allows for the complete replacement of surface ligands, resulting in QDs that retain their photoluminescence and exhibit improved electronic passivation.^22^

Thiocyanate has been demonstrated as a successful agent for the passivation of II–VI nanocrystals,^24^ perovskite solar cells,^25–29^ and recently perovskite nanocrystals.^30–32^ When the surface is treated with thiocyanate, nanocrystals present significantly improved quantum yield while maintaining structure and morphology.^31–36^ The literature is split in assigning the exact mechanism underlying the enhanced emission in treated nanocrystals. Koscher et al. associate it with thiocyanate scavenging of excess lead from NC surfaces.^31,32^ Others claim that thiocyanate should bind to bromine vacancies on the surfaces, fulfilling those traps and subsequently increasing PLQY and ambient stability.^37^ Here, we bring a result that supports the hypothesis that thiocyanate binds to the crystal surface. In addition, we do not find evidence suggesting Pb removal or the formation of Pb­(SCN)_2_.

Furthermore, in the design of such surface treatment, the need to use an ionic solid salt at an organic liquid interface is limited by the solubility of the salt in the colloidal suspension. In this work, we demonstrate that we can develop a colloidally compatible surface treatment by optimizing a procedure based on an ionic liquid of thiocyanate. Our process is adopted from perovskite solar cell literature and adjusted to accommodate colloidal suspensions.^28^ We thus overcome critical inherent limitations that arise from the ionic nature of thiocyanate salts and the sensitivity of halide perovskites to a polar environment. Our process enhances the solubility of thiocyanate by working at the liquid–liquid interface of UAT and the CsPbBr_3_ nanocrystal colloid. The ionic liquid UAT enables a more significant loading of the NCs without structural degradation.

Our analysis presents atomic resolution evidence of thiocyanate binding to NC surfaces, which results in improved PLQY and stability and slows ion migration in treated CsPbBr_3_ nanocrystals.

## Experimental Methods

### Materials

Cs_2_CO_3_ (99.5% Sigma-Aldrich), lead acetate trihydrate (Pb­(CH_3_COO)_2_·3H_2_O, 99.99% Sigma-Aldrich) benzoyl bromide (C_6_H_5_COBr, 97% Sigma-Aldrich), octadecene (ODE, 90%, Sigma-Aldrich), oleic acid (OA, 90%, Sigma-Aldrich), oleylamine (OLA, 70%, Sigma-Aldrich), hexanes (>99%, Aldrich), *n*-octane (>99%, Aldrich), ammonium thiocyanate (99.5%, Sigma-Aldrich), urea (>90% Sigma-Aldrich), lead bromide (PbBr_2_ 99.5% Sigma-Aldrich), lead iodide (PbI_2_ 99.9% Sigma-Aldrich).

### Nanocrystal Synthesis

Sixteen milligram portion of cesium carbonate, 76 mg of lead acetate trihydrate, 0.3 mL of OA, 1 mL of OLAM, and 5 mL of ODE were loaded into a 25 mL 3-neck round-bottom flask and degassed under vacuum for 1 h at 120 °C. The temperature was then increased to 170 °C under nitrogen flow, and 78 μL of benzoyl bromide was injected. The reaction mixture was immediately cooled in an ice water bath to room temperature, at which point it could be disconnected from the gas manifold. Five milliliters of hexane was added to the crude NC solution in aliquots to wash the reaction vessel, and the resulting mixture was centrifuged for 10 min at 8000 rpm. The supernatant was discarded, and the precipitate was redispersed in 5 mL of hexane and subsequently centrifuged for another 5 min at 4000 rpm. The supernatant was collected. Typical concentration was determined and ranged between 1 and 10 μM. Adapted from Imran et al.^38^

### UAT Synthesis

Both urea and ammonium thiocyanate were oven-dried at 100 °C for 24 h and stored in a vacuum desiccator until ready to be used. UAT was produced via gentle heating and stirring at 50 °C for 3 h with a 1.4:1 molar ratio of urea to ammonium thiocyanate. Afterward, the two salts will form a deep eutectic solvent and remain as a liquid at room temperature. It is kept away from moisture, as it tends to hydrate and partially recrystallize. Adapted from Yu et al.^28^

### UAT Treatment

UAT is immiscible in nonpolar organic solvents typical for the suspension of perovskite nanocrystals such as hexane and toluene; as a result, it sinks to the bottom of the vial. A typical treatment consists of two passivation steps. In each treatment step, 1% by volume of UAT was added.

After synthesis during the first washing step, 50 μL of UAT is added to 5 mL of crude resuspension and is pulsed briefly on the vortexer. A second centrifugation step will follow in which the treated perovskite NC solution can be safely decanted.

In the second step, 10 μL of UAT is added to 1 mL of nanocrystal solution. The sample is briefly pulsed on a vortexer to ensure that mixing has occurred. The treated solution is decanted via pipet, leaving the small insoluble UAT bead at the bottom to be discarded.

Samples were then characterized accordingly.

### UV–Vis Absorption, PL, and Excitation Measurements (PLE)

For optical measurements, 200 μL of the sample solution was injected into a 96-well microplate or 4 mL of the sample solution in a Take-3 holder with a quartz cuvette and measured in a Synergy H1 hybrid multimode reader. The samples were irradiated by using a xenon lamp (Xe900). Both the 5 mL and 200 μL sample solutions were prepared in 1:20 dilution NCs:hexane. The 1:20 dilution ratio was carefully selected to optimize the optical density of the nanocrystal solution and minimize inner filtering effects, particularly reabsorption and self-quenching. These effects can significantly distort the accuracy of UV–vis and PL measurements, especially in highly absorbing samples. By maintaining the sample within the linear detection range of the instrument, this dilution ensures a more reliable and representative spectroscopic characterization.

### Lifetime, Photoluminescence Quantum Yield (PLQY), and Kinetic Emission Measurements

Lifetime, photoluminescence quantum yield (PLQY), and kinetic emission characterizations were performed by using the Edinburgh FLS1000 photoluminescence spectrometer. All of the samples were loaded into a quartz cuvette. The lifetime measurements were performed with time-correlated single-photon counting (TCSPC) mode and conducted using an efficient pulse laser (EPL) of 405 nm wavelength (EPL405). Both the lifetime and kinetic measurements were performed with a cuvette holder inside the spectrophotometer. The PLQY measurements were performed with an integrating sphere holder inside the spectrometer. Both the PLQY and the kinetic measurements were performed using a xenon lamp excitation source. 2–4 mL of samples were prepared in a 1:20 dilution and further diluted if necessary.

### X-ray Diffraction (XRD) Characterizations and Two-Dimensional Grazing Incidence Wide-Angle X-ray Diffraction (2D-GIWAX)

The NC’s solution was drop-cast onto a rectangular micro slide glass substrate (76 mm × 26 mm) for θ–2θ measurements or sliced micro slide glass substrate (10 mm × 10 mm) for 2D-GIWAX measurements. Measurements were taken using a Rigaku Smart-Lab 9 kW high-resolution X-ray diffractometer equipped with a rotating anode X-ray source. We used a 1.54Å (Cu Kα) wavelength. We performed θ–2θ measurements with a 2θ range of 10–60°, using a Ge-2X200 monochromator. We performed 2D-GIWAX measurements using a Hy-Pix3000 2D detector and a 2D-SAXS/WAXS (reflection) attachment with a reflection beam stopper and aperture slit.

### Transmission Electron Microscopy (TEM) Characterization

One drop of a dilute nanocrystal solution in hexane (1:50 dilution) was cast onto a TEM grid (carbon film side, 300-mesh copper grid). The samples were observed in TEM mode with a Thermo Fisher/FEI Tecnai G2 T20 S-Twin LaB_6_ TEM operated at 200 keV.

For characterization at the atomic scale, a Thermo Fisher/FEI Titan Themis double Cs-Corrected HR-S/TEM operated at 200 keV was used.

The microscope is equipped with a Dual-X detector (Bruker Corporation, USA) for energy-dispersive X-ray spectroscopy (EDS) elemental mapping and a Gatan Quantum ER965 dual-EELS detector (Gatan) for electron energy loss spectroscopy (EELS) analysis.

EDS maps were acquired, postprocessed, and analyzed using the Velox software (Thermo Fisher).

### Fourier-Transform Infrared (FTIR) Spectroscopy

Infrared spectroscopy was performed with sample solutions placed between two 2 mm-thick CaF_2_ windows separated with a 60 μm Teflon spacer. FTIR spectra of SCN^–^/OA/OLAM and Pb­(SCN)_2_/OA/OLAM solutions in octane were measured on a Nicolet iS10 (Thermo Scientific), whereas the SCN^–^/NCs solution was measured on Tensor 27 (Bruker) spectrometers. Each spectrum was averaged for 500 scans with 4 cm^–1^ resolution; all data were collected at room temperature (22 °C).

### Density Functional Theory Simulations

Density Functional Theory calculations have been performed using the GPAW code^39^ and the Atomistic Simulation Environment (ASE) package.^40^ All structures have been relaxed in the framework of the generalized gradient approximation (GGA) using the PBEsol exchange–correlation functional.^41^ The calculations have been performed in the plane-wave mode with an energy cutoff of 500 eV and a k-point uniform density of 3 k-points/Å, Γ-centered. The structures were relaxed until the residual energies and forces were below 10^–5^eV and 0.05 eV/Å, respectively.

## Results

In this work, we demonstrate a method to improve perovskite surface treatment by increasing the thiocyanate solubility in nonpolar solvents using ionic liquid UAT, as demonstrated in Figure 1a. The eutectic liquid is an ionic solvent comprised of two or three substances that exhibit self-association and possess a melting point lower than that of each individual substance. They are formulated through the combination of a compatible hydrogen bond acceptor (HBA) and a hydrogen bond donor (HBD).^42,43^ Regarding potential chemical reactions between urea and ammonium thiocyanate in comparison with pure ammonium thiocyanate, it is hypothesized that elevating the urea/ammonium thiocyanate ratios could enhance the ammonium and urea bonding. Consequently, reducing the strength of the Coulombic interaction of the SCN^–^ and enhancing the thiocyanate freedom in solution. Yu et al. demonstrated that urea/ammonium thiocyanate ratios of 1.5 and above do not align with this hypothesis. Given that, we used a molar ratio of 1.4, reported by Yu et al., to yield the optimal cell performance in cesium lead triiodide solar cells. For the optimization of the liquid–liquid UAT-based surface treatment, we evaluated how ligand concentration in the colloidal suspension affects the thiocyanate solubility. For halide perovskite nanocrystals, labile surface ligands form a highly dynamic ligand shell,^44,45^ which actively participates in anion exchange reactions.^46,47^ In addition, the inability of polar thiocyanate to dissolve in the nonpolar colloidal suspension further limits the reaction rate and the surface treatment efficacy. Figure 1b shows a solubility calibration curve of the thiocyanate solubility as a function of the volume fraction of ligands. This curve was used to optimize and develop a colloidal-compatible surface process. The construction of the calibration curve is based on FTIR measurements conducted on samples containing UAT with different volume fractions of oleylamine and oleic acid. The observed increase in the ligand-fixated thiocyanate 2050 cm^–1^ peak area is optimized. Marked in a shaded green bar is the point at which the addition of ligands will not significantly increase UAT solubility but may harm the CsPbBr_3_ NCs.

**Figure 1:**
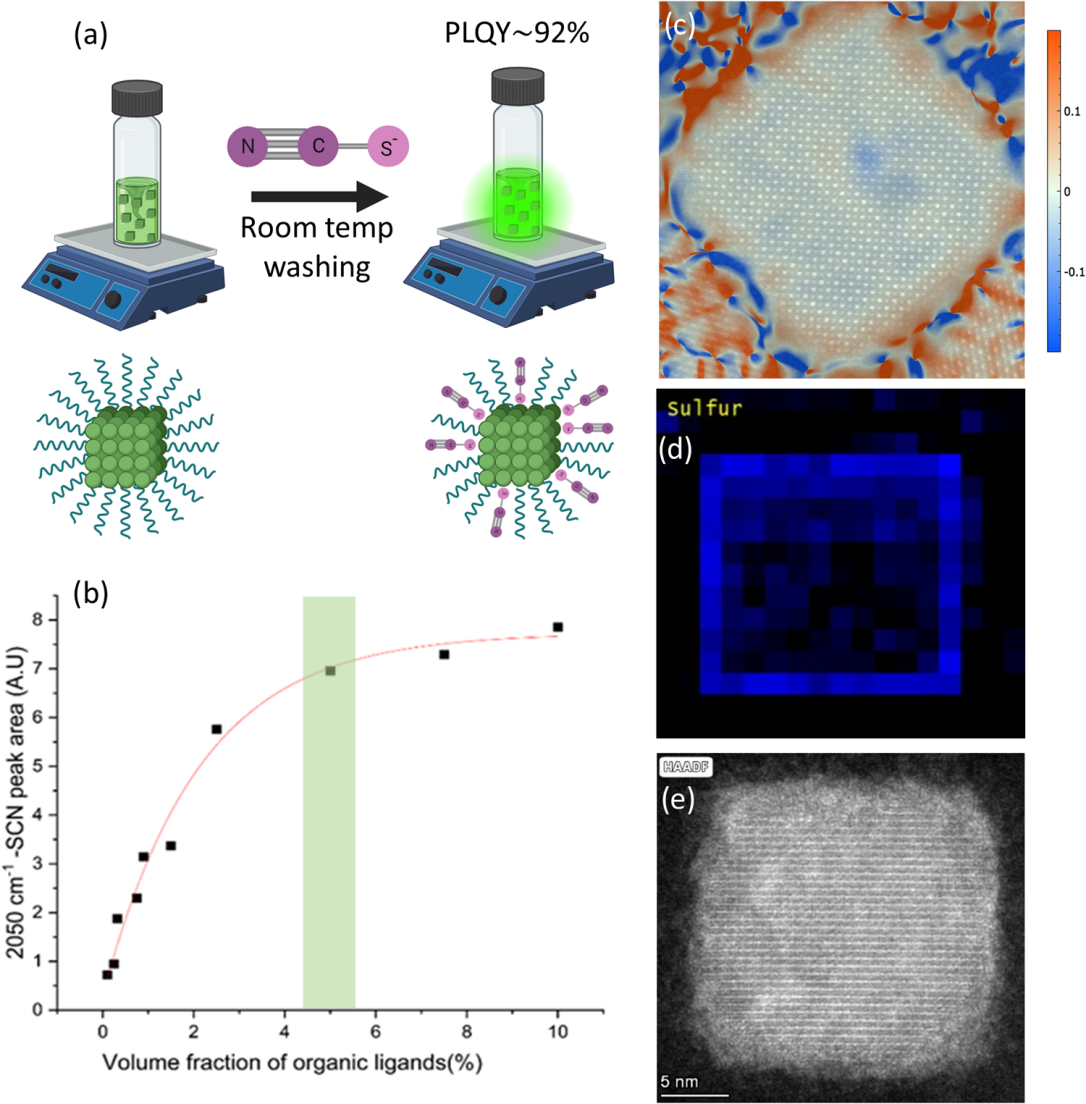
(a) Surface treatment illustration (created with BioRender.com). (b) Solubility saturation curve of SCN^–^ FTIR peak area at 2050 cm^–1^ as a function of ligand volume fraction. The FTIR data is available in Figure S1. (c) Dilatation map generated by GPA analysis of HAADF micrograph acquired at 200 keV. (d) EELS sulfur signal mapping acquired at 60 keV. (e) HAADF micrograph of the perovskite NC used to acquire EELS mapping.

As the concentration of ligands increases, the peak area of ligand-fixated thiocyanate also increases until a plateau is reached at a 10% volume fraction of ligands. This synergistic effect of the presence of oleic acid and oleylamine together with thiocyanate emphasizes the ligands’ role in the solubility of thiocyanate from UAT. However, they also may be related to the binding and passivation process of thiocyanate to the surface due to their dynamic adsorption–desorption nature.^48^ We estimate the optimal value in which UAT is properly dissolved, yet the amount of free ligand is minimal, a volume fraction of about 5%.

We now delve into the mechanism of the UAT surface treatment optimized for colloidal suspensions. We can assume that thiocyanate attaches to the surface due to the essential role played by the ligands oleylamine and oleic acid since the coordination modes of thiocyanate in an electrolytic ionic liquid are complex.^49–51^ This is also evident from IR spectroscopy studies.^52^ Our further demonstrated understanding is based on structural and spectroscopic analysis. Beyond the conventional XRD and TEM, this will be discussed later. In this study, we pushed the experimental boundaries of halide perovskite nanocrystal characterization by using TEM and STEM.

We compared HAADF-STEM micrographs of treated and untreated NCs. We then conducted geometrical phase analysis (GPA)^53^ in order to measure the structural impact of the surface treatment on the crystals. This technique allows for the analysis of strain and dilatation in crystals by comparing the phase of reference, unstrained lattice planes, to those observed in an image. This comparison involves extracting specific Fourier components from the image using a mask, calculating phase differences, and then determining displacements and strain fields by analyzing these phases for multiple vectors (see Figure S2). By leveraging these phase differences, GPA can provide information on deformations, including strain, rotation, and dilatation, confirming the structural homogeneity and mechanical properties of materials. Figure 1c exhibits surface-treated NC HAADF-STEM micrographs with a dilatation map showing positive dilatation of the crystal structure at the edges. This dilatation is depicted in red, framing all of the edges of the treated crystal. We then analyzed the dilated lattice and estimated the expanded lattice parameter at the NC surface to be 3% larger than the unperturbed interior of the NC. We hypothesize that this expansion is because molecules are binding directly to Pb cations, effectively acting as pseudohalides and filling the Br^–^ vacancy for the UAT-treated NCs. This is in line with previous reports by us and others on surface-bound SCN^–^.^54^ The dilated shell may be a result of replacing smaller halides (Br^–^) with bulkier SCN^–^ leading to an increased steric load at lattice sites, effectively forcing the observed expansion. A second critical difference between the halide and the pseudohalide is charge redistribution on the crystal surface since, for the pseudohalide charges, they are delocalized across multiple atoms, which weakens the attraction between the Pb^+^ and anion, enabling the observed expansion.

A second approach to characterize the SCN^–^-treated NCs was to conduct electron energy loss spectroscopy (EELS) measurements. The main challenge in these types of characterizations is avoiding degradation and damage to the crystal. For this experiment, by selecting a low acceleration voltage of 60 keV and short exposures, we were able to complete EELS mapping without any apparent electron beam structural damage to the nanocrystals. Contrary to energy-dispersive spectroscopy (EDS), EELS allows retrieval of information from low energy peaks, making a full spectral scan unnecessary, thus reducing the total exposure time. In our case, there is an energy overlap between Pb and S peaks (Mα and Kα, respectively); EELS allows better energy resolution than EDS, enabling us to resolve the cases of sulfur presence in the nanocrystal shell (see Figures S4–S8).

From the various data sets we acquired (S8), our EELS measurements suggest that thiocyanate is bound preferably to the surfaces of the nanocrystal, forming a shell-like signature of the fitted energy loss signal. Figure 1d,e exhibits surface-treated NC HAADF-STEM micrographs and the fitted EELS sulfur elemental map, proving sulfur is primarily attached to the particle surface and is not homogeneously distributed in the crystals’ volume. In some of the data sets, we witness variations in the sulfur ratio between crystal edges and faces (see Figure S8). This statistical variance probably indicates inhomogeneities in UAT coverage on the NC surface and not deeper penetration of thiols into the interior of the nanocrystals.^46^

In the ongoing debate on the mechanism behind thiocyanate surface passivation of CsPbBr_3_ NCs, we highlight this result in agreement with the hypothesis of surface-bound thiocyanate that substitutes for surface bromine vacancies. According to the hard–soft acid–base theory, large and polarizable ions will tend to attach to each other.^55^ Thiocyanate is considered a pseudohalide anion with a (−1) formal charge. Both lead and UAT share large ionic radii, which are easily polarizable. Therefore, we expect the sulfur of thiocyanate to bind to Pb and, as a result, will passivate bromide vacancies on the NC’s surface. To further support this model, we calculated the energy required to passivate a bromide vacancy on the surface of CsPbBr_3_ (Figure 2a) via Density Functional Theory (DFT) simulations. From the pristine 2 × 2 × 5 (001) surface of CsPbBr_3_, 0.74 eV is required to form a Br-vacancy (calculated versus Br_(s)_). The surface is then passivated by SCN^–^ occupying the Br-vacancy spot, i.e., bonding to the dangling bond of the Pb. This process is energetically favorable (−2.96 eV vs pristine surface of CsPbBr_3_ and HSCN_(g)_). The resulting highly favorable energetic difference of −2.22 eV explains the efficient thiocyanate surface passivation of CsPbBr_3_ NCs.

**Figure 2:**
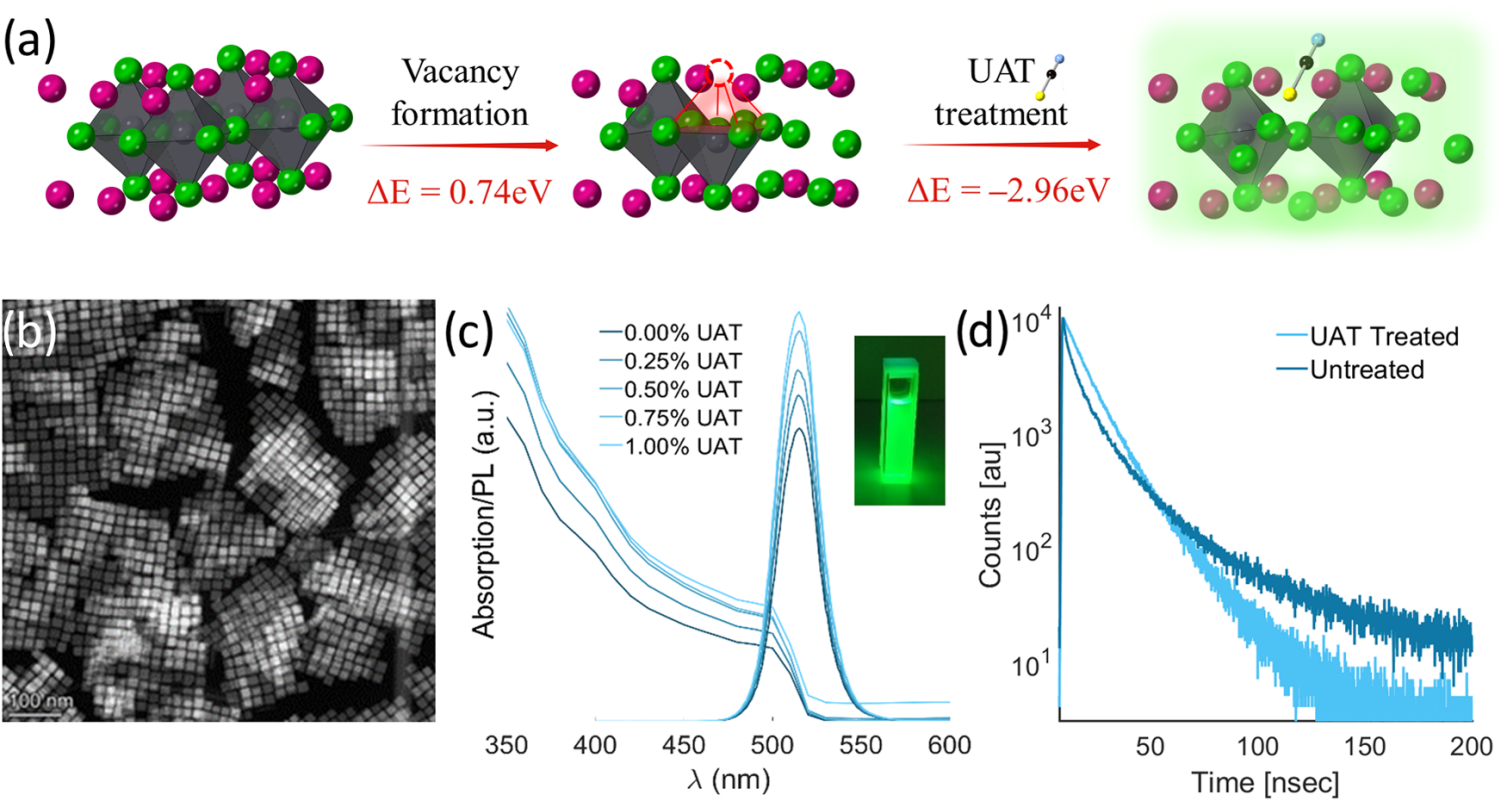
(a) Illustration and DFT energy calculations of bromide vacancy formation and SCN^–^ molecule filling the vacancy during the UAT treatment (graphic design using CrystalMaker, CrystalMaker Software Ltd.).^56^ (b) HAADF-STEM of CsPbBr_3_-treated nanocrystals. (c) PL emission demonstrating increased emission intensity with volumetric addition. (d) PL lifetime of treated and untreated nanocubes.

As part of the optimization process, we tested various variations of the treatment. We optimized for the highest PLQY and demonstrated that two rounds with volumetric addition of 1% UAT (v/v) are the most efficient, as shown in Table 1 and Figure 2c. Further support of the thiocyanate–bromide vacancy passivation is found in time-resolved spectroscopic studies. We show that the lifetime decay varies after the UAT treatment (Figures 2d and S9 and Table S1); this result is similar to previously published UAT papers.^32,35^ We attribute these changes to the passivation of shallow trapped states on the NC’s surface caused by bromide vacancies. Such surface passivation results in a PLQY increase. Along with the decay profile, it is dominated by faster band-edge recombination, indicating that carriers now recombine more efficiently via radiative pathways.

**Table 1:** Photoluminescence Quantum Yield Measurements of Treated and Untreated Samples

treatment	% PLQY
as synthesized	44 ± 2
adding 1% UAT	86 ± 4
adding 1% UAT and cleaning	87 ± 4
adding another 1% and cleaning	92 ± 4

In terms of structural characterization, no visible difference in the perovskite crystal structure could be detected in both XRD and TEM characterization (Figure 3). TEM micrographs from treated and untreated samples exhibit similar morphologies (Figure 3a,d) and structures (Figure 3b,c,e,f). The TEM electron diffraction (Figure 3b,c) of the untreated NCs and the treated NCs’ FFT pattern (Figure 3f) indicate the integrity of the perovskite structure post-treatment. The HAADF-STEM (Figure 2b) and TEM (Figure 3a) micrographs demonstrate that the overall CsPbBr_3_ lattice structure is maintained, supporting mainly a surface effect from our UAT-based process. The X-ray diffractogram also clearly shows that no deviation from the original structure was observed before and after treatment (Figure 3h). While all peaks agree with the calculated XRD pattern, there is a split peak at *Q*/2π = ∼1.75 [1/nm] (marked with a gray rhombus in Figure 3h). We note in passing that this peak arises via collective diffraction effects from the formation of superlattices, which naturally self-assemble in the drying process of samples; see detailed analysis by Toso et al.^57^

**Figure 3:**
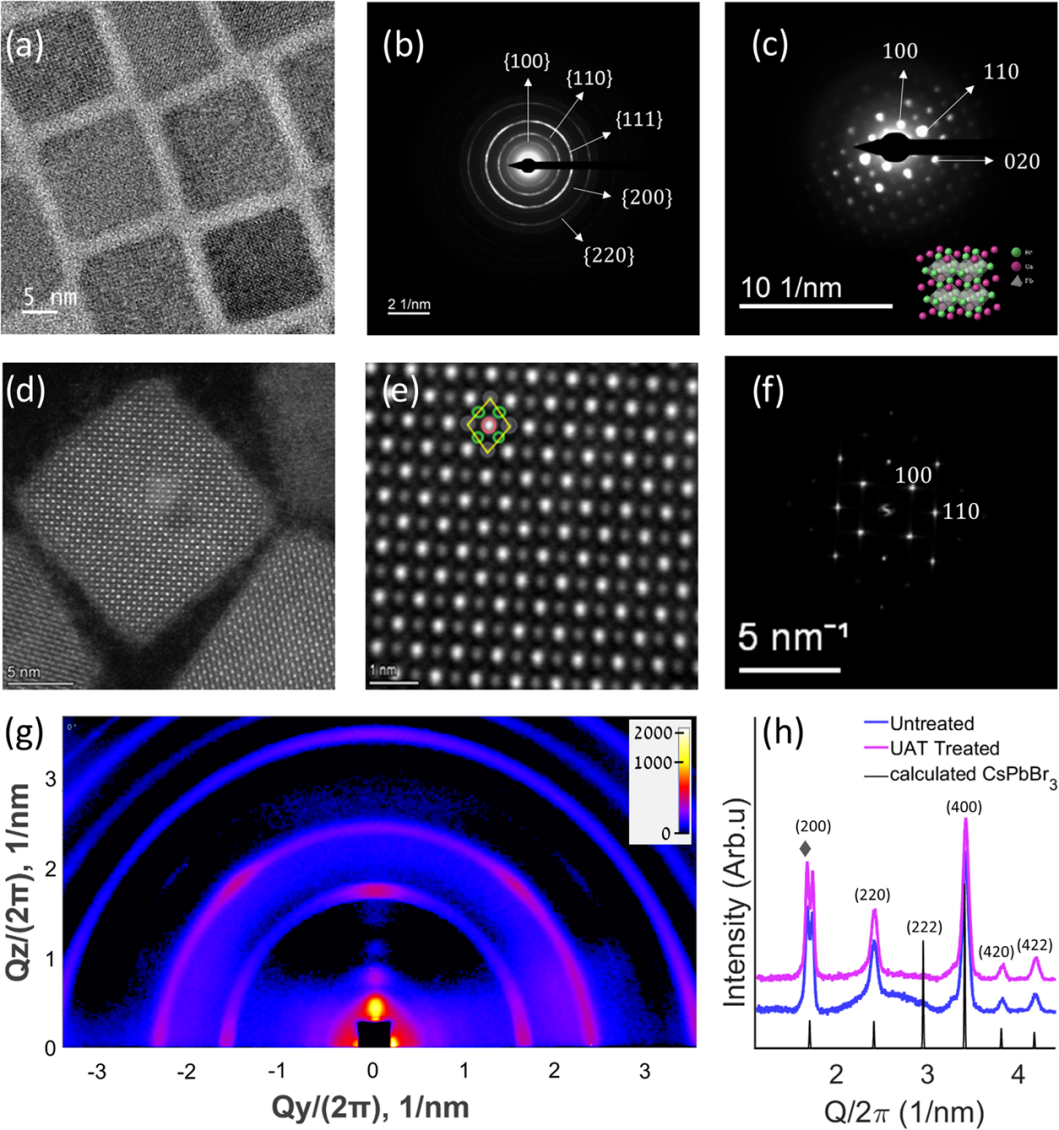
(a) TEM micrograph of pristine CsPbBr_3_ NCs. (b) TEM ring pattern and (c) single-crystal diffraction of pristine CsPbBr_3_ (graphic design using CrystalMaker, CrystalMaker Software Ltd.).^56^ (d, e) HAADF-STEM micrographs of CsPbBr_3_-treated NCs, with (f) the corresponding FFT. (g) 2D-GI-WAXS mapping of treated sample. CsPbBr_3_ planes appeared as arcs. Peaks of the higher order of periodicity are present in the vertical direction as dots for *Q*/2π < ∼1.75 [1/nm].^58^ Analysis of 1D projections of (g) is available in the Supporting Information. (h) XRD patterns of untreated (blue) and UAT-treated (pink) samples. Calculated CsPbBr_3_ peaks are added (black). The split of the (200) peak (marked with a gray rhombus) is due to a higher order of periodicity.

Such superlattice ordering is further confirmed using 2D-GI-WAXS (Figure 3g) by out-of-plane peaks (bright spots for *Q*_y_/2π < ∼1.75 [1/nm] reflect such ordering (Figures 3g and S10). For higher *Q*/2π, we observed peaks related to the internal perovskite structure as arcs (Figure S10a,d).^58,59^ Notice the intensity along the arcs enhanced at specific angles. This indicates the main orientation of the NCs in the sample (additional analysis of the 2D-GI-WAXS is available in Figure S10).

Past experiments demonstrated that halide perovskite with inorganic shells is more stable to ionic substitution.^60^ Now, when we see evidence for alteration of the outermost shell of atoms in SCN^–^ surface-treated NCs, a similar reasoning is valid. We therefore study the anion exchange kinetics of treated NCs in order to test their stability to anion substitution. Understanding this effect is of great advantage for further advancement in applications and devices.^61,62^ For the experiment, we tested the anion exchange rate by following the change in the emission central wavelength with time. Figure 4a shows the in situ ion exchange experiment (details in the Supporting Information). In both single and double-treated samples, we see that the emission spectrum (Figure 4b,c) shows a monotonic dependence with time which we interpret as halide composition.^63^ In Figure 4d, the central emission wavelength is plotted against time, demonstrating that within the same reaction time, the untreated samples undergo a significantly greater change of composition. This is true both for the iodine (redshift) and chlorine (blueshift) exchange, which is consistent with the literature.^47,64,65^

**Figure 4:**
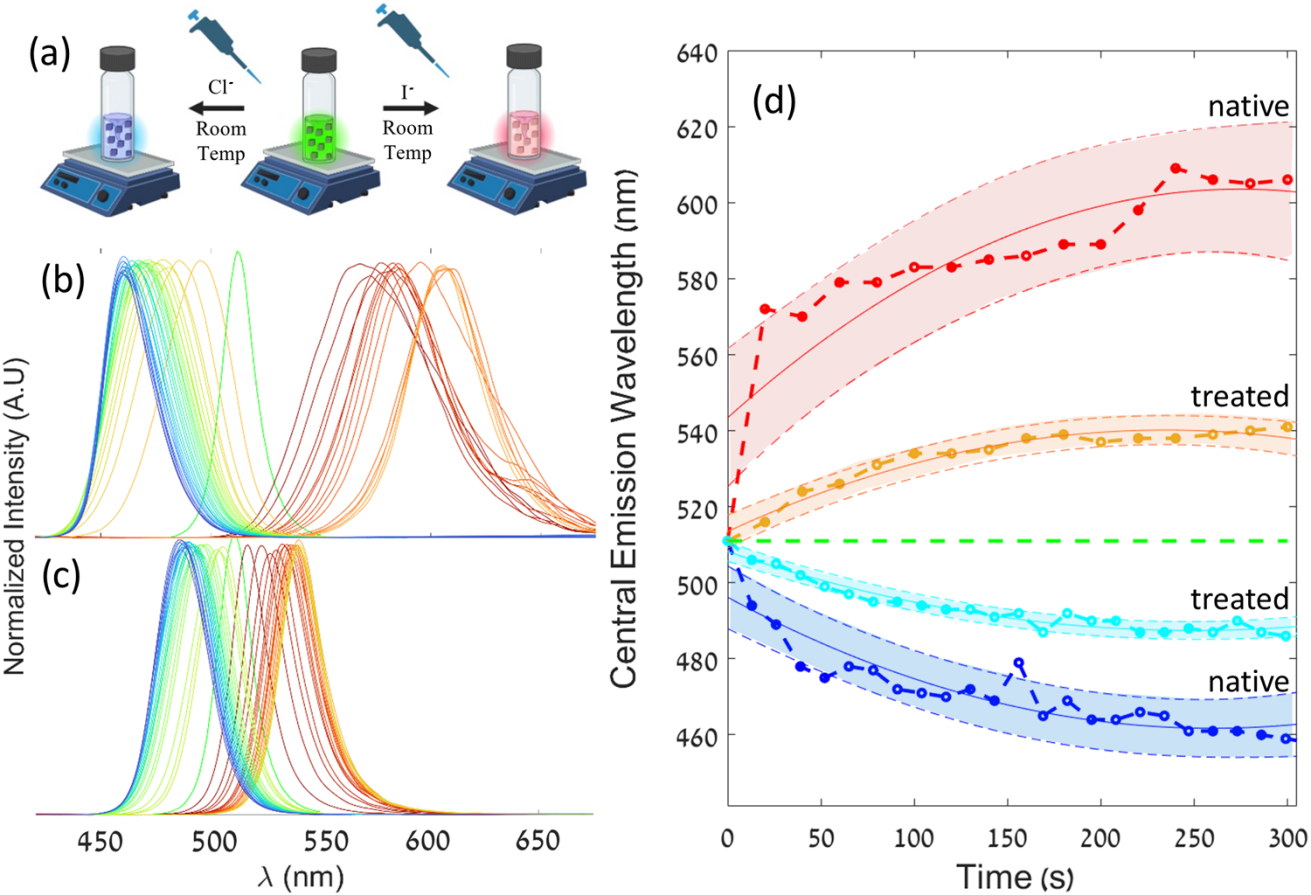
(a) Illustration of anion exchange experiment with I^–^ and Cl^–^ (created with BioRender.com). In situ anion exchange PL measurements of the (b) pristine and (c) twice UAT-treated samples. Scans were taken every ∼20 s to monitor the progress of the anion exchange. (d) Central emission wavelength progression during the anion exchange experiments.

We attribute the difference between the treated and untreated samples to the thiocyanate on the surface that dilates the outer shell, affecting the diffusion and substitution of anions. We hypothesize that thiocyanate is bound to Pb and replaces a bromide vacancy on the perovskite surface. Since the bromide vacancies are key to the diffusion and migration of the replacing halides (either chlorine or iodine), the thiocyanate serves as a barrier. The observed differences between the treated and untreated samples may additionally arise from ligand shell rigidity modifications. Rigid ligand architectures create energy barriers that suppress halide diffusion by restricting anion mobility through steric hindrance and enhanced surface passivation.^66^ By extension, the concentration of thiocyanate on the surface is anticorrelated with the anion exchange rate, as demonstrated by the added stability to ion exchange shown in the second treatment compared to the first (see Figure S11).

We thus show that not only are the treated NCs brighter due to bromide vacancy passivation, but they are also more stable to anion diffusion. Such properties are crucial for advancing halide perovskite NCs, which are in great need of increasing lasting stability and improving optoelectronic properties.^67^

## Conclusion

The presented surface treatment using UAT at the liquid–liquid interface of the nanocrystal suspension was optimized for colloidal CsPbBr_3_ NCs. Through enhanced solubility at the liquid–liquid interface, we can passivate the NC surfaces without damage to the polar moieties of the halide perovskite. In the debate regarding the mechanism behind the PLQY enhancement effect, we confirm that thiocyanate is surface-bound and bring evidence in the form of a dilated shell and EELS, which explain the functional improvement in PLQY and anion stability. Future studies should be expanded to differentiate the role of morphologies, compositions, and more complex heterostructures on their optoelectronic properties and colloidal stability study.

## Supplementary Material



## Notes

Materials; methods descriptions, including syntheses, optical, XRD, TEM, HAADF-STEM, EDS characterization, and EELS characterization; EELS spectra and data; GPA analysis; additional lifetime decay parameters; additional XRD patterns; and additional anion exchange emission spectra. (PDF)
