# Supplementary material for: Liquid–Liquid Interface-Based Thiocyanate Surface Treatment for Bright and Stable CsPbBr3 Nanocrystals
Source: Chem Mater. 2025 May 25;37(11):4178–86. doi: 10.1021/acs.chemmater.5c00803 (PMC12159974; doi:10.1021/acs.chemmater.5c00803)
Supplement: Supplementary file 1 [file cm5c00803_si_001.pdf]

Supporting Information

Liquid-Liquid Interface-Based Thiocyanate Surface Treatment for Bright and Stable $CsPbBr_3$ Nanocrystals

Rachel Lifer[1], Nathan Rafisiman[1,2], Saar Shaek[1], Arghyadeep Basu[2,3], Yaron Kauffmann[1], Nicholas G. Pavlopoulos[2], Ivano E. Castelli[4], Lev Chuntonov[2,3], Yehonadav Bekenstein[1,3,5]*

Affiliations:

[1]Department of Materials Science and Engineering, Technion – Israel Institute of Technology, 32000 Haifa, Israel.

[2]Department of Chemistry, Technion – Israel Institute of Technology, 32000 Haifa, Israel.

[3]The Solid-state institute, Technion – Israel Institute of Technology, 32000 Haifa, Israel.

[4]Department of Energy Conversion and Storage (DTU Energy), Technical University of Denmark, Anker Engelunds Vej 301, 2800 Kongens Lyngby, Denmark.

[5]The Resnick Sustainability Center for Catalysis, Technion – Israel Institute of Technology, Technion City, Haifa, 3200009, Israel.

*Corresponding author. Email: bekenstein@technion.ac.il

- Rachel Lifer, Nathan Rafisiman, and Saar Shaek contributed equally.

S1. Experimental Methods

Materials: Cs_2CO_3 (99.5% Sigma-Aldrich), Lead acetate trihydrate ($Pb(CH_3COO)_2 \cdot 3H_2O$, 99.99% Sigma-Aldrich) Benzoyl Bromide (C_6H_5COBr, 97% Sigma-Aldrich) Octadecene (ODE, 90%, Sigma-Aldrich), Oleic Acid (OA, 90%, Sigma-Aldrich), Oleylamine (OLA, 70%, Sigma-Aldrich), Hexanes (>99%, Aldrich), n-Octane (>99%, Aldrich), Ammonium Thiocyanate (99.5%, Sigma-Aldrich), Urea (>90% Sigma-Aldrich), Lead Bromide ($PbBr_2$ 99.5% Sigma-Aldrich), Lead Iodide (PbI_2 99.9% Sigma-Aldrich), Lead Chloride ($PbCl_2$ 99.9% Sigma-Aldrich).

UV−Vis Absorption, PL, and excitation measurements (PLE):

For optical measurements, 200μL of the sample solution was injected into a 96-well microplate or 4mL of the sample solution in a Take-3 holder with a quartz cuvette and measured in a Synergy H1 hybrid multimode reader. The samples were irradiated using a xenon lamp (Xe900). The 4mL and 200μL sample solutions were prepared in 1:20 dilution nanocrystals-hexane.

Lifetime, photoluminescence quantum yield (PLQY), and kinetic emission measurements:

Lifetime, photoluminescence quantum yield (PLQY), and kinetic emission characterizations were performed using the Edinburgh FLS1000 photoluminescence spectrometer. All the samples were loaded into a quartz cuvette. The lifetime measurements were performed with time-correlated single-photon counting (TCSPC) mode and conducted using an efficient pulse laser (EPL) of 405nm wavelength (EPL405). The lifetime and kinetic measurements were performed with a cuvette holder inside the spectrophotometer. The PLQY measurements were performed with an integrating sphere holder inside the spectrometer. The PLQY and the kinetic measurements were performed using a xenon lamp excitation source. 2-4mL samples were

prepared in 1:20 dilution and further diluted if necessary. For the kinetic measurements, the scans were taken every ∼20 seconds to monitor the progress of the anion exchange.

X-ray diffraction (XRD) characterizations and two-dimensional grazing incidence wide-angle X-ray diffraction (2D-GIWAX):

The NC's solution was drop-cast onto a rectangular micro slide glass substrate (76mmx26mm) for θ-2θ measurements or sliced micro slide glass substrate (10mmx10mm) for 2D-GIWAX measurements. Measurements were taken using a Rigaku Smart-Lab 9kW high-resolution X-ray diffractometer equipped with a rotating anode X-ray source. We used a 1.54Å (Cu Kα) wavelength. We performed θ-2θ measurements with a 2θ range of 10°-60°, using a Ge-2x220 monochromator. We performed 2D-GIWAX measurements using Hy-Pix3000 2D detector and a 2D-SAXS/WAXS (reflection) attachment with a reflection beam stopper and aperture slit.

Transmission electron microscopy (TEM) characterization:

One drop of a dilute nanocrystal solution in hexane (1:50 dilution) was cast onto a TEM grid (carbon film side, 300-mesh copper grid). The samples were observed in TEM mode with a Thermo Fisher/FEI Tecnai G^2 T20 S-Twin LaB_6 TEM operated at 200keV.

A Thermo-Fisher/FEI Titan Themis double Cs-Corrected HR-S/TEM operated at 200KeV was used for characterization at the atomic scale and elemental analysis.

The microscope is equipped with a Dual-X detector (Bruker Corporation, USA) for energy-dispersive X-ray spectroscopy (EDS) elemental mapping and a Gatan Quantum ER965 dual-EELS detector (Gatan, USA) for electron energy loss spectroscopy (EELS) analysis.

EDS maps were acquired, post-processed, and analyzed using the Velox software (Thermo-Fisher, USA).

Fourier-transform infrared (FTIR) Spectroscopy:

Infrared spectroscopy was performed with sample solutions placed between two 2-mm-thick CaF_2 windows separated by a 60µm Teflon spacer. FTIR spectra of SCN^-/OA/OLAM and $Pb(SCN)_2$/OA/OLAM solutions in octane were measured on Nicolet iS10 (Thermo Scientific), whereas SCN^-/NCs solution was measured on Tensor 27 (Bruker) spectrometers. Each spectrum was averaged for 500 scans with 4cm^{-1} resolution; all data were collected at room temperature (22°C).

Density Functional Theory Simulations:

Density Functional Theory calculations have been performed using the GPAW code[1] and the Atomistic Simulation Environment (ASE) package[2]. All structures have been relaxed in the framework of the generalized gradient approximation (GGA) using the PBEsol exchange-correlation functional[3]. The calculations have been performed in the plane-wave mode with an energy cutoff of 500eV and a k-point uniform density of 3 k-points/Å, Γ-centered. The structures were relaxed until the residual energies and forces were below 10^{-5}eV and 0.05eV/Å, respectively.

Additional results

S2- Surface and elemental analysis:

We used FTIR measurements to detect if the SCN^- is bound to the NC's surface. We followed the thiocyanate's nitrile bond stretch (2000-2100 cm^{-1}) in different chemical environments.

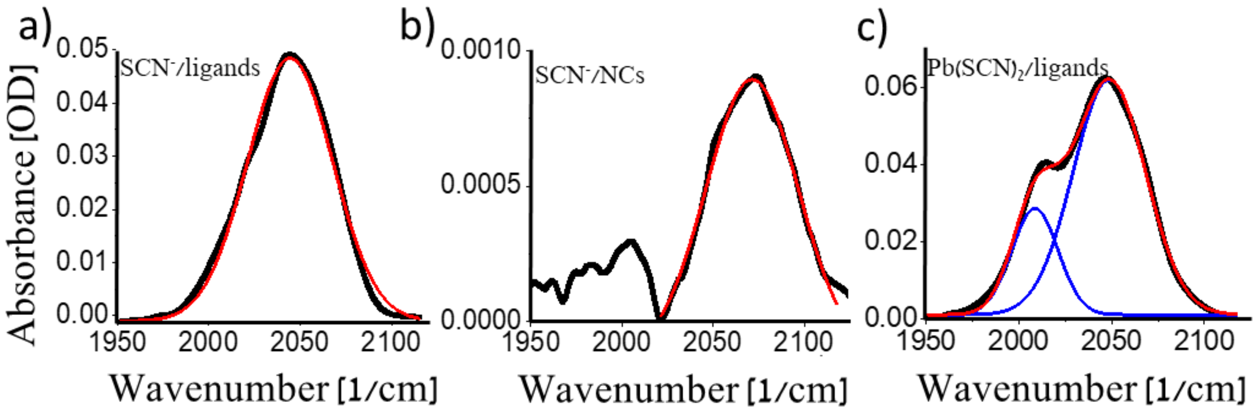

Figure S1: FTIR spectrum of (a) UAT ligand-assisted solvation in octane, (b) $CsPbBr_3$ NCs treated with UAT, and (c) $Pb(SCN)_2$.

Without the NCs, the SCN^- stretching frequency in solution (with OA and OLA ligands) is ν_{sol}=2046cm^{-1} (Figure S8a). On NCs, the SCN^- transition is blue-shifted to ν_{NC}=2071cm^{-1} and has a broad full-width-half-maximum (FWHM) bandwidth, $\Delta\nu_{NC}$=60cm^{-1} (Figure S8b). The spectral peak FWHM is similar to the FWHM of the SCN^-/ligands sample, $\Delta\nu_{sol}$=55cm^{-1}. To understand if the blue shift originates from Pb-SCN bonding, we measured the IR spectra of the $Pb(SCN)_2$ complex (Figure S8c). The IR spectra of $Pb(SCN)_2$ feature two broadband transitions at $\nu_{Pb(SCN)2,1}$=2008cm^{-1} and $\nu_{Pb(SCN)2,2}$=2048cm^{-1}. These peaks are distinct from the ν_{NC}, indicating that the blue-shifted peak of the SCN^-/NCs sample is related to surface-bound SCN^- and not to the Pb-SCN bond.

We have examined the HAADF STEM micrograph of treated perovskite, which was performed via Geometrical Phase Analysis (GPA) analysis using an open-source application, Strain++[4]. GPA is a technique that allows for the analysis of strain and dilatation in crystals by comparing the phase of reference, unstrained lattice planes, to those observed in an image. This process involves extracting specific Fourier components from the image using a mask, calculating phase differences, and subsequently determining displacements, strain fields, and dilatation. In this analysis, a high-resolution HAADF STEM micrograph of the UAT-treated perovskite particle (Figure S1a) was used as input. The Fast Fourier Transform (FFT) of the micrograph was calculated to identify periodicities in reciprocal space, and two distinct g-vectors were selected for analysis, highlighted by red and blue circles (Figure S1b). To analyze the red g-vector, a mask was applied to isolate its Fourier component (Figure S1c). An inverse FFT of this masked component produced a phase image corresponding to the red g-vector (Figure S1d). The phase image was then normalized to remove linear gradients and refine the analysis, as shown in Figure S1e. This process was repeated for the blue g-vector. Finally, combining the displacement fields calculated from both g-vectors allowed us to generate a dilation map with very high sensitivity to changes due to the Fourier transform properties. (Figure S1f), revealing local expansion among the surface of perovskite particle.

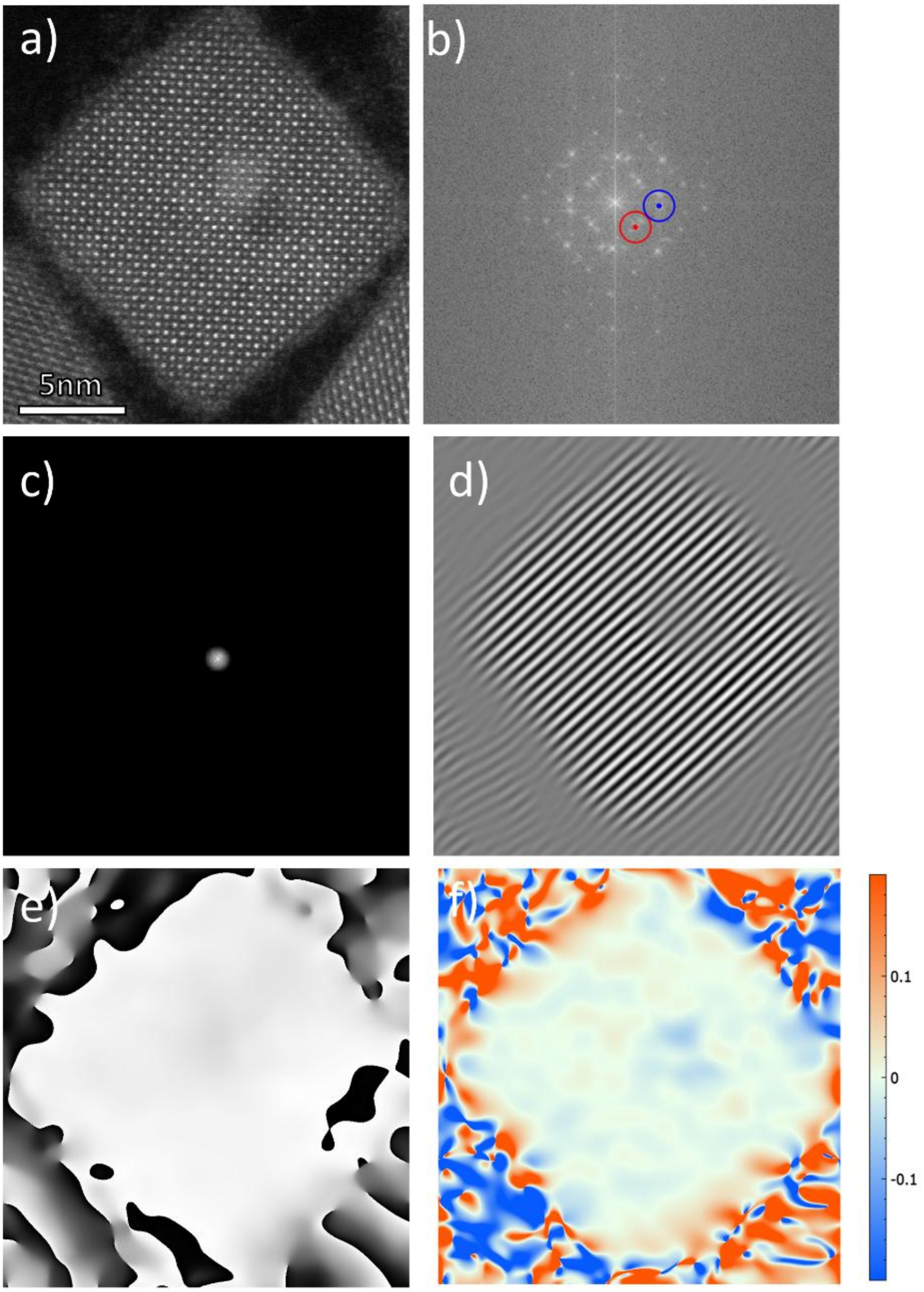

Figure S2: a) high resolution HAADF STEM micrograph of UAT treated particle for GPA analysis. b) FFT of the micrograph with two selected g vectors. c) masked FFT of the red g vector. d) phase image of the red g vector. e) normalized phase. f) resulting dilatation map after applying those steps for the blue vector as well.

A control experiment where HAADF STEM micrograph of untreated particle was analyzed is displayed. Applying the same procedure on similar micrographs did not indicate any detectable structural dilatation data is shown in figure S3.

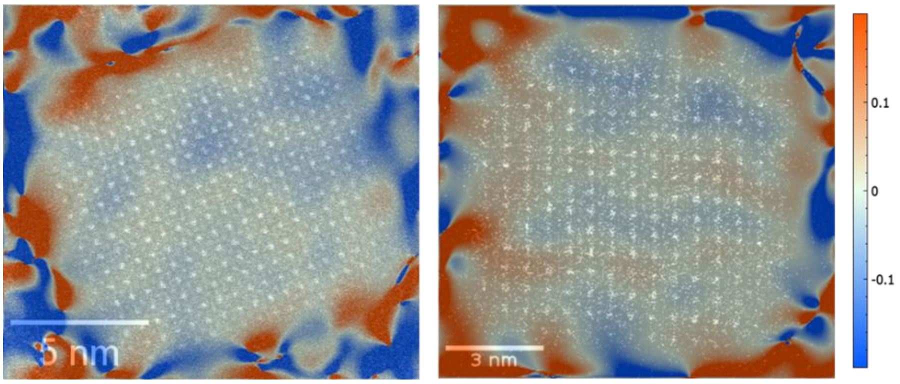

Figure S3: a) and b) GPA of HAADF STEM micrograph of untreated particle does not indicate any dilatation of the outer surfaces.

The elemental analysis includes EDS, EELS mapping, and FTIR.

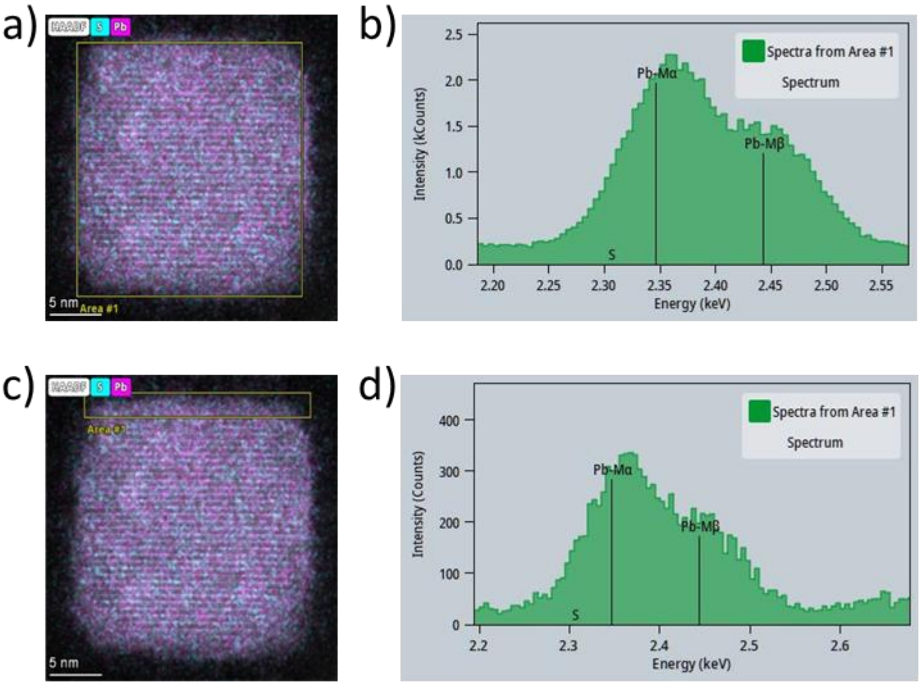

	Entire nanocrystal	Edge
S (%at)	0.00	0.00
Br (%at)	57.89	49.26
Cs (%at)	19.58	21.28
Pb (%at)	22.53	29.45

Figure S4: (a) Selected area of the entire nanocrystal. (b) EDS spectra of the nanocrystal area in (a). (c) Selected area of an edge (a slice of the surface). (d) EDS spectra of the edge area in (c).

As seen in Figure S4(a,b) and the table below, the EDS measurements from the entire nanocrystal enable us to ensure the ABX_3 perovskite stoichiometry. However, due to the spectral overlap of lead and sulfur and the energy resolution scale of EDS, it is impracticable to detect the presence of sulfur or get a precise measure of its presence. This convolution is intrinsic to the energy levels present in these elements. However, the atomic fractions change by selecting an edge of the particle (a single surface slice), and we no longer have the ABX_3 perovskite stoichiometry. There is an increase in the lead signal (that overlaps with the sulfur signal) and a decrease in the bromine content, indicating surface elemental changes due to the post-treatment on the nanocrystal's surface. However, the fundamental issue of the Pb/S overlap remains.

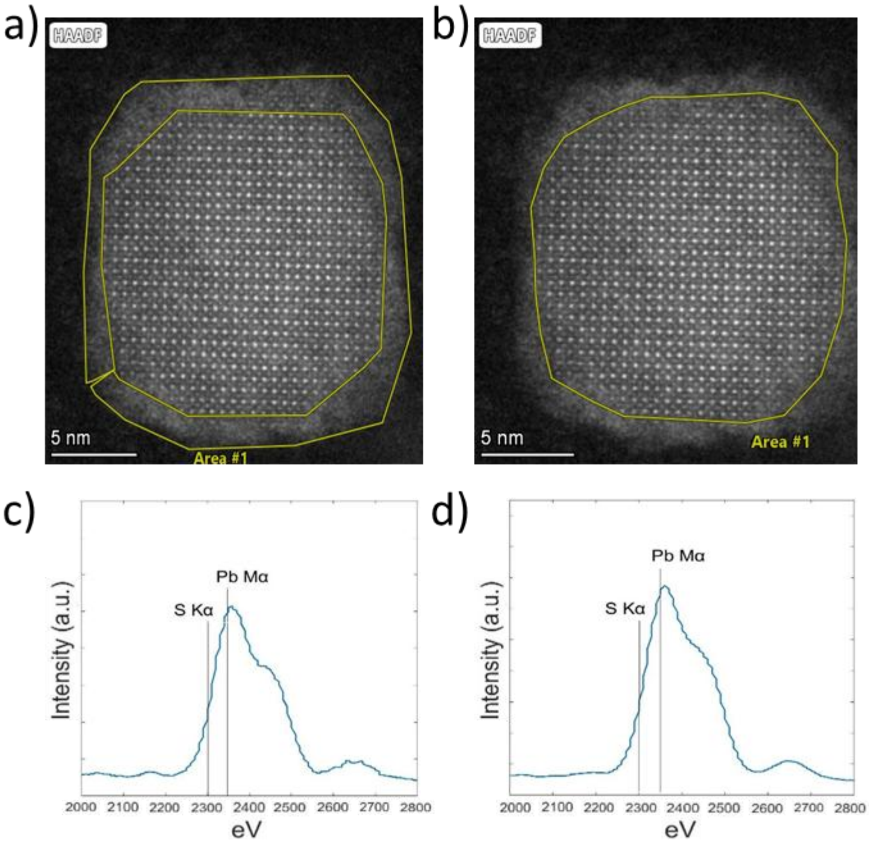

Figure S5: Selected area EDS mapping of both the surface (a) and core (b) spectral overlap between lead and sulfur in both areas (c) (d), respectively.

The EDS mapping of the surface and core confirms the overlap between lead and sulfur, making it impossible to deconvolute the spectral peaks associated with their energies. This gave some indication that the sulfur peak was buried underneath the lead. To resolve this, we needed a finer resolution and moved to EELS.

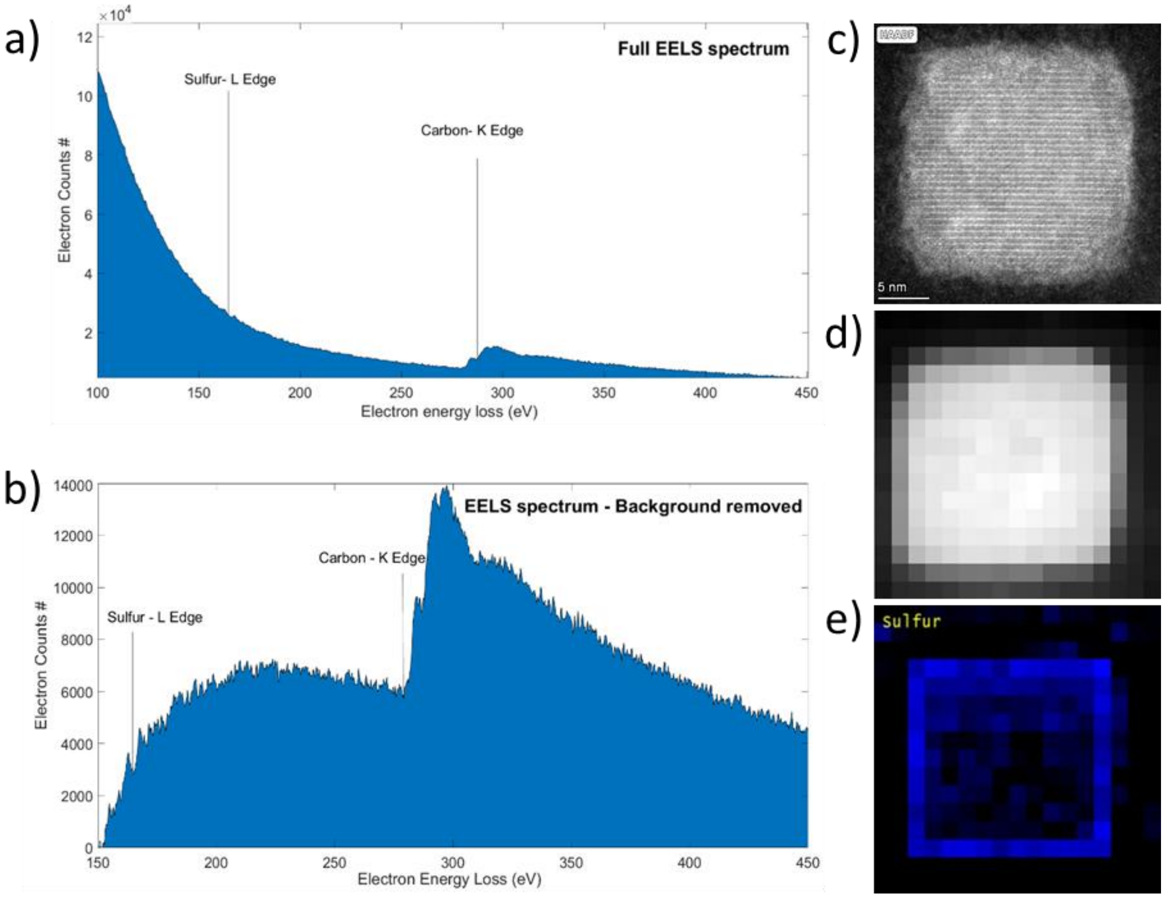

Figure S6: (a) Full EELS spectrum with background intact, (b) with background removed to visualize the sulfur L23. (c) HAADF-STEM micrograph of the perovskite nanocrystal used to acquire EELS spectra. d) EELS total signal mapping, and (e) sulfur signal mapping. EELS was obtained at 60 keV.

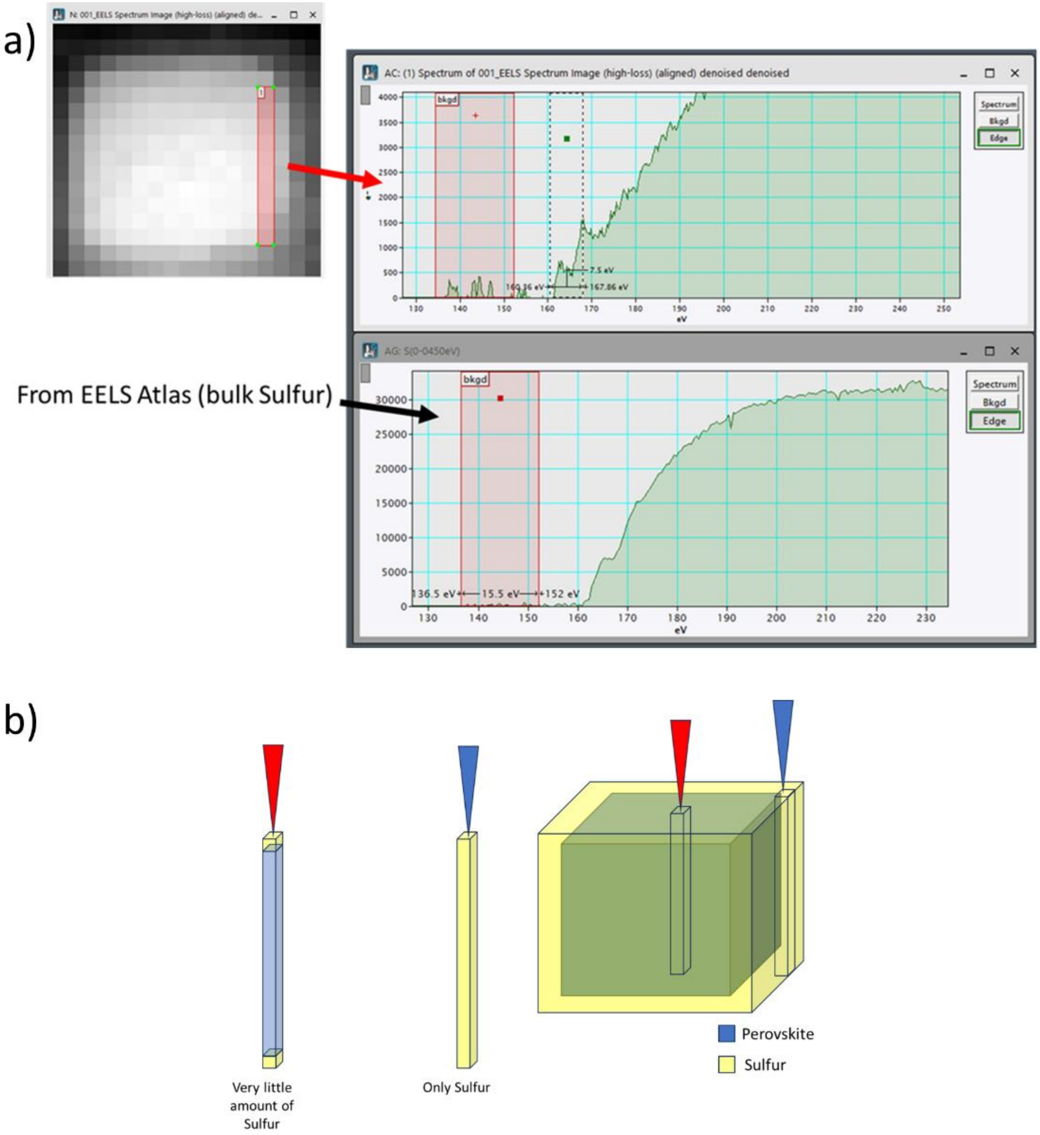

Figure S7: (a) EELS spectrum of the UAT treated sample and a reference spectrum from bulk Sulfur (based on the EELS atlas from Gatan). (b) illustration of the particle sulfur coverage corresponding to the EELS scans.

Electron energy loss spectroscopy (EELS) enables separating lead and sulfur signals with a much finer resolution, from 10^2 eV to 1 eV. This gives a better indication of sulfur presence in a lead-containing background. In this experiment, we were able to use a lower acceleration voltage so as not to damage the sample via beam damage and increase the likelihood of scattering effects. This allows us to directly collect information from the lower L23 edge of the element we are interested in, bypassing the issue of the overlapping peaks altogether. Figure

S6e, with the sulfur color filter applied, clearly shows that sulfur is bound predominantly to the surface of the perovskite.

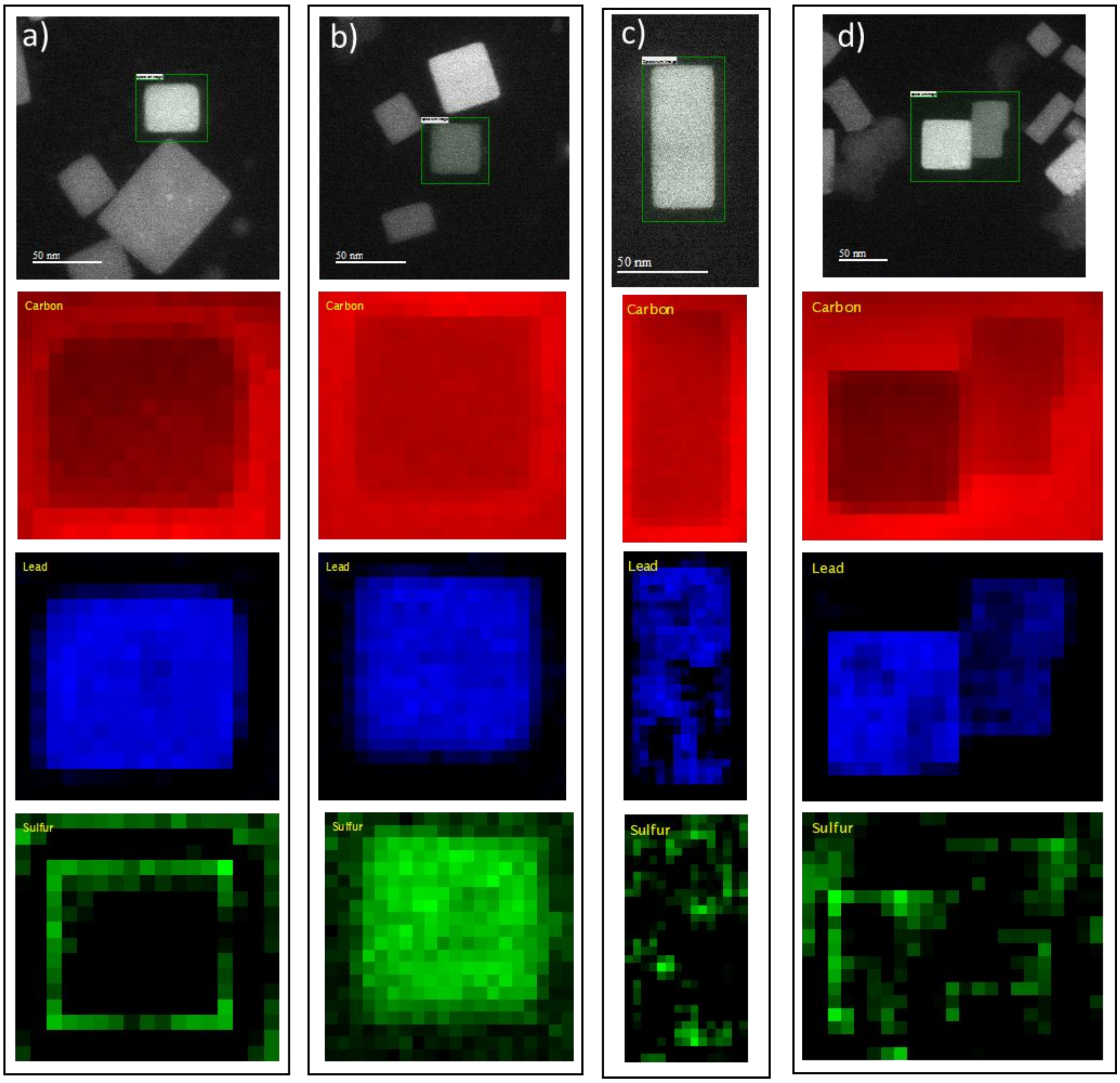

Figure S8: a-d) EELS mapping of UAT treated particles, in red carbon K mapping, in blue lead N mapping, and in green Sulfur $L_{2,3}$. (a) (c)and (d) indicates surface located sulfur. (b) is an example of a particle in which sulfur signal is found further inside the crystal.

Figure S8 shows a selection of EELs measurements in the majority of samples the sulfur signal is indeed surface located. It should be mentioned that in some cases Sulfur signal was throughout the particle. But in this case the sulfur signal intensity in higher at the surface.

S3. Optical analysis:

The optical analysis includes lifetime decay of UAT treated and untreated samples.

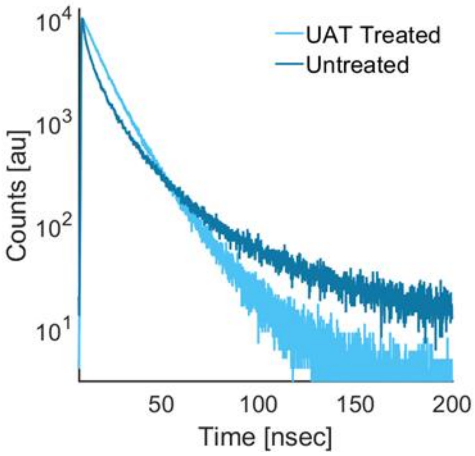

Figure S9: PL lifetime of treated and untreated NCs.

As seen in Figure S9, the lifetime decay changes after the UAT treatment. We believe this is related to bromide vacancies (trap states) passivation on the surface of the NCs. Detailed analysis is shown in Table S1.

Table S1: lifetime fitting parameters of untreated and UAT treated samples

	Untreated		UAT treated	
	value	Rel [%]	value	Rel [%]
B_1	5178±30	29.91	6588±121	45.95
B_2	2830±27	70.09	2698±128	55.05
τ_1 [nsec]	3.94±0.04		8.54±0.09	
τ_2 [nsec]	16.90±0.09		17.1±0.2	
$\tau_{\text{Amp-Weighted Avg}}$ [nsec]	8.55		11.02	
K_{rad} [1/sec]	$5.15\cdot10^7$		$7.68\cdot10^7$	
K_{nonrad} [1/sec]	$6.55\cdot10^7$		$1.39\cdot10^7$	

Time-resolved photoluminescence (TRPL) studies reveal distinct lifetime changes post-treatment. These shifts are attributed to the thiocyanate passivation of bromide vacancies, which suppresses shallow trapped states on the nanocrystal surface, redirecting carriers toward radiative recombination as seen in the higher K_{rad} of the treated sample.

S4. Structural analysis:

The structural analysis includes XRD and 2D-GI-WAXS.

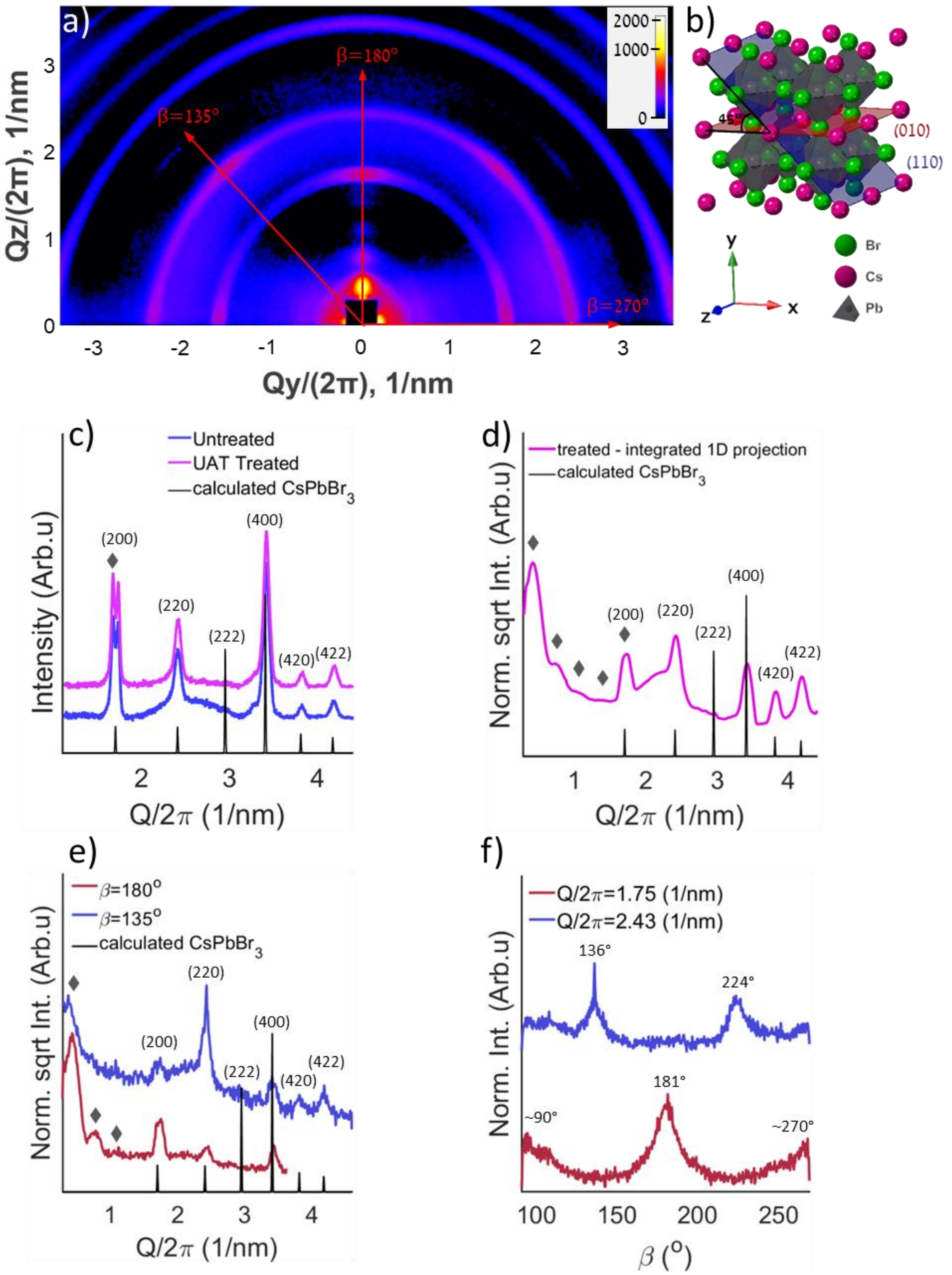

Figure S10: (a) 2D-GI-WAXS mapping of treated sample. $CsPbBr_3$ planes appeared as arcs. Peaks of a higher order of periodicity are present in the vertical direction (β=180°C) and

lower $Q_z/2\pi$ as dots[5,6]. (b) Calculated $CsPbBr_3$ model, with a marked (010) plane (red) and (110) plane (blue) and an angle of 45° between them (graphic designed using CrystalMaker®, CrystalMaker Software Ltd)[7]. (c) XRD patterns of untreated (blue) and UAT-treated (pink) samples. The split of the (200) peak is due to a higher order of periodicity[8]. (d) Integrated 1D projection, (e) 1D projections from the directions of β=180° and β=135°, and (f) 1D projections of the intensity dependence on the direction (β angles) for Q/2π=1.75 [1/nm] and Q/2π=2.43 [1/nm], of the 2D-GI-WAXS mapping in (a). Calculated $CsPbBr_3$ peaks (black) and assembly periodicity peaks (gray rhombus) are added in (c-e). The X-axis in (d) and (e) is presented in the square root of the intensity for emphasis.

Using the 2D-GI-WAXS, we conclude the nanocubes are self-assembled and primarily oriented in the (100) direction. As presented in Figure S10a, for Q/2π<~1.75 [1/nm], there are peaks (dots) apparent in the out-of-plane direction of the measurement (β=180°). These peaks are related to high-order self-assembly and stacking of particles, one above the other[6,8]. These peaks (marked with gray rhombus) are also present in the XRD measurement shown in Figure S10c and the 1D projections in S10d and S10e. From Q/2π≥~1.75 [1/nm], the peaks (arcs/ half rings) in Figure S10a are related to the $CsPbBr_3$ perovskite structure (model of the structure is presented in S10b) and in agreement with the XRD pattern in S10c. For clarity, an integrated 1D projection of S10a is shown in S10d. Both the perovskite structure pattern and the stacking peaks are visible in the integrated projection.

From the different intensities along the arcs (rings) in S10a, we get a deeper understanding of the particles' orientation and stacking. We used 1D projections of S10a in the specific β angles (directions) of 180° and 135° (Figure S10e) and 1D projections of the intensity dependence on β angles for Q/2π=1.75 [1/nm] and Q/2π=2.43 [1/nm] (Figure S10f). In the β=180° direction (out-of-plane), the most intense perovskite structure peak is the (200), and the stacking peaks are also visible. Looking at 45° from the out-of-plane direction (at β=135°), the most intense perovskite structure peak is the (220). This agrees with the 45° angle between the (010) and (110), shown in Figure S10b. The intensity along the Q/2π=1.75 [1/nm] arc (Figure S10f) is highest at β=~180°, and also relatively high at β=~90°, ~270°. The intensity along the Q/2π=1.75 [1/nm] arc (Figure S10f) is highest at β=~135°,~225°. These results explain the

split peak at $Q/2\pi<\sim1.75$ [1/nm] in Figure S10c and confirm most of the nanocubes are oriented and self-assembled in the (100) direction.

S5. Kinetic study:

The Kinetic study includes in-situ anion exchange emission spectra of pristine, UAT treated and twice UAT treated samples. In these experiments Lead halide solutions (PbI_2 or $PbCl_2$) were added to both treated and untreated NC solutions. Lead halide solutions preparations were adapted from Kosher et al.[9] In situ photoluminescence (PL) measurements were recorded every 20 seconds to track spectral shifts indicative of halide exchange over a total duration of 300 seconds.

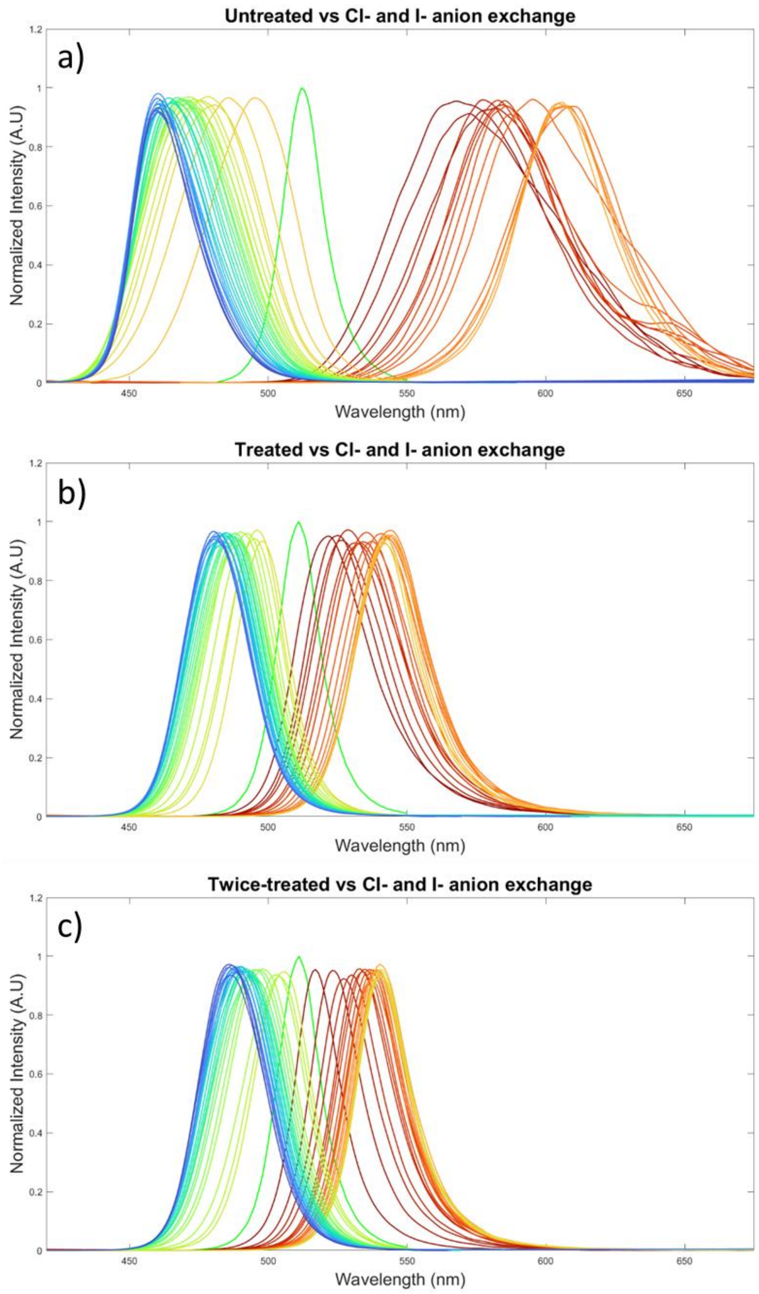

Figure S11: In situ anion (I^- and Cl^-) exchange PL measurements of the (a) pristine, (b) UAT treated and (c) twice UAT treated samples. All the kinetic experiments start from the same native $CsPbBr_3$ solution sample (peak at ∼512nm).

As seen from Figure S11 the anion exchange rate with both I^- and Cl^- slows down after the UAT treatment, especially with the twice treatment. We believe the thiocyanate replace bromide vacancy as a pseudohalide, and therefore as a diffusion barrier to halide exchange through the surface of the NCs.

References

(1) Enkovaara, J.; Rostgaard, C.; Mortensen, J. J.; Chen, J.; Dułak, M.; Ferrighi, L.; Gavnholt, J.; Glinsvad, C.; Haikola, V.; Hansen, H. A.; Kristoffersen, H. H.; Kuisma, M.; Larsen, A. H.; Lehtovaara, L.; Ljungberg, M.; Lopez-Acevedo, O.; Moses, P. G.; Ojanen, J.; Olsen, T.; Petzold, V.; Romero, N. A.; Stausholm-Møller, J.; Strange, M.; Tritsaris, G. A.; Vanin, M.; Walter, M.; Hammer, B.; Häkkinen, H.; Madsen, G. K. H.; Nieminen, R. M.; Nørskov, J. K.; Puska, M.; Rantala, T. T.; Schiøtz, J.; Thygesen, K. S.; Jacobsen, K. W. Electronic Structure Calculations with GPAW: A Real-Space Implementation of the Projector-Wave Method. *Journal of Physics: Condensed Matter* 2010, *22* (25), 253202.

(2) Hjorth Larsen, A.; JØrgen Mortensen, J.; Blomqvist, J.; Castelli, I. E.; Christensen, R.; Dułak, M.; Friis, J.; Groves, M. N.; Hammer, B.; Hargus, C.; Hermes, E. D.; Jennings, P. C.; Bjerre Jensen, P.; Kermode, J.; Kitchin, J. R.; Leonhard Kolsbjerg, E.; Kubal, J.; Kaasbjerg, K.; Lysgaard, S.; Bergmann Maronsson, J.; Maxson, T.; Olsen, T.; Pastewka, L.; Peterson, A.; Rostgaard, C.; SchiØtz, J.; Schütt, O.; Strange, M.; Thygesen, K. S.; Vegge, T.; Vilhelmsen, L.; Walter, M.; Zeng, Z.; Jacobsen, K. W. The Atomic Simulation Environment—a Python Library for Working with Atoms. *Journal of Physics: Condensed Matter* 2017, *29* (27), 273002.

(3) Perdew, J. P.; Ruzsinszky, A.; Csonka, G. I.; Vydrov, O. A.; Scuseria, G. E.; Constantin, L. A.; Zhou, X.; Burke, K. Restoring the Density-Gradient Expansion for Exchange in Solids and Surfaces. 2008.

(4) Hÿtch, M. J.; Snoeck, E.; Kilaas, R. Quantitative Measurement of Displacement and Strain Fields from HREM Micrographs. *Ultramicroscopy* 1998, *74* (3), 131–146.

(5) Kobayashi, S.; Inaba, K. GI-XRD Measurements of Thin Film Samples Using a 2D Detector and 2D-SAXS/WAXS Attachment. *Rigaku Journal (ISSN 2187 9974), Volume 35, No. 1,5-8.*

(6) Dror, S.; Khalfin, S.; Veber, N.; Lang, A.; Kauffmann, Y.; Koifman Khristosov, M.; Shechter, R.; Pokroy, B.; Castelli, I. E.; Bekenstein, Y. Transformations of 2D to 3D Double-Perovskite Nanoplates of Cs2AgBiBr6 Composition. *Chemistry of Materials* 2023, *35* (3), 1363–1372.

(7) Palmer, D. C. Visualization and Analysis of Crystal Structures Using CrystalMaker Software. *Z Kristallogr Cryst Mater* 2015, *230* (9–10), 559–572.

(8) Toso, S.; Baranov, D.; Filippi, U.; Giannini, C.; Manna, L. Collective Diffraction Effects in Perovskite Nanocrystal Superlattices. *Acc Chem Res* 2023, *56* (1), 66–76.

(9) Koscher, B. A.; Bronstein, N. D.; Olshansky, J. H.; Bekenstein, Y.; Alivisatos, A. P. Surface- vs Diffusion-Limited Mechanisms of Anion Exchange in CsPbBr3 Nanocrystal Cubes Revealed through Kinetic Studies. *J Am Chem Soc* 2016, *138* (37), 12065–12068.
